# Assessing the Formation of Purine Lesions in Mitochondrial DNA of Cockayne Syndrome Cells

**DOI:** 10.3390/biom12111630

**Published:** 2022-11-03

**Authors:** Chryssostomos Chatgilialoglu, Marios G. Krokidis, Annalisa Masi, Sebastian Barata-Vallejo, Carla Ferreri, Barbara Pascucci, Mariarosaria D’Errico

**Affiliations:** 1 Istituto per la Sintesi Organica e la Fotoreattività, Consiglio Nazionale delle Ricerche, Via P. Gobetti 101, 40129 Bologna, Italy; 2 Center for Advanced Technologies, Adam Mickiewicz University, 61–614 Poznań, Poland; 3 Institute of Nanoscience and Nanotechnology, N.C.S.R. “Demokritos”, Agia Paraskevi Attikis, 15310 Athens, Greece; 4 Institute of Crystallography, Consiglio Nazionale delle Ricerche, Monterotondo Stazione, 00015 Rome, Italy; 5 Departamento de Ciencias Químicas, Facultad de Farmacia y Bioquimíca, Universidad de Buenos Aires, Junin 954, Buenos Aires CP 1113, Argentina; 6 Department of Environment and Health, Istituto Superiore di Sanità, Viale Regina Elena 299, 00161 Rome, Italy

**Keywords:** mitochondrial and nuclear DNA damage, 5′,8–cyclopurines, 8–oxo–dG, gamma radiolysis, hydroxyl radical, isotope dilution LC–MS/MS, cockayne syndrome

## Abstract

Mitochondrial (mt) DNA and nuclear (n) DNA have known structures and roles in cells; however, they are rarely compared under specific conditions such as oxidative or degenerative environments that can create damage to the DNA base moieties. Six purine lesions were ascertained in the mtDNA of wild type (wt) CSA (CS3BE–wtCSA) and wtCSB (CS1AN–wtCSB) cells and defective counterparts CS3BE and CS1AN in comparison with the corresponding total (t) DNA (t = n + mt). In particular, the four 5′,8–cyclopurine (cPu) and the two 8–oxo–purine (8–oxo–Pu) lesions were accurately quantified by LC–MS/MS analysis using isotopomeric internal standards after an enzymatic digestion procedure. The 8–oxo–Pu levels were found to be in the range of 25–50 lesions/10^7^ nucleotides in both the mtDNA and tDNA. The four cPu were undetectable in the mtDNA both in defective cells and in the wt counterparts (CSA and CSB), contrary to their detection in tDNA, indicating a nonappearance of hydroxyl radical (HO^•^) reactivity within the mtDNA. In order to assess the HO^•^ reactivity towards purine nucleobases in the two genetic materials, we performed γ–radiolysis experiments coupled with the 8–oxo–Pu and cPu quantifications on isolated mtDNA and tDNA from wtCSB cells. In the latter experiments, all six purine lesions were detected in both of the DNA, showing a higher resistance to HO^•^ attack in the case of mtDNA compared with tDNA, likely due to their different DNA helical topology influencing the relative abundance of the lesions.

## 1. Introduction

Molecular oxygen (O_2_) is used for the production of reactive oxygen species (ROS) that are involved in the signaling pathways of various basal and adaptive physiological responses controlling organism homeostasis [1,2,3,4]. However, ROS are also responsible for a variety of pathological processes, as their overproduction contributes to biomolecule damage, which has been linked with the etiology of various diseases [4,5,6]. Under physiological conditions, most human resting cells experience ca. 5% oxygen tension; however, the [O_2_] gradient occurring between the extracellular environment and mitochondria, where oxygen is consumed by cytochrome c oxidase, results in a significantly lower [O_2_] exposition of mitochondria [7,8,9]. It is estimated that up to 1% to 5% of the consumed mitochondrial oxygen is converted to ROS [10]. In 1972, Harman proposed that mitochondria were the primary source of cellular free radicals, and were thus responsible for the free–radical–based ageing process [11].

Human mitochondrial DNA (mtDNA) is a circular molecule of ~16.5 kb and must be compacted in order to fit within a mitochondrion [12,13]. mtDNA exists in a compacted DNA–protein complex known as the mitochondrial nucleoid that may be protective towards the source of mitochondrial free radicals [14]. As cellular ROS are produced by the mitochondrial respiratory chain, in the form of superoxide, much attention has been focused on the putative role of ROS in mitochondrial mutagenesis [15]. mtDNA such as nuclear DNA (nDNA) is highly susceptible to ROS, and is easily oxidized to accumulate DNA modifications [16,17,18]. Increased oxidative damage in mtDNA has been associated with neurological degeneration, inflammasomes, tumorigenesis, and malignant progression [19,20]. Among the mtDNA repair pathways, the base excision repair (BER) pathway has been extensively characterized to remove some of oxidative DNA damage in the mitochondria as efficiently as in the nuclei [17]. Implications of other repair pathways remain unclear, although the absence of nucleotide excision repair (NER) in the mitochondria is well documented [17]. Indeed, despite the permanent exposure to ROS and the less protective pathways in the mitochondria, it is not clarified how the integrity of genetic information is maintained in this compartment [16,17,18].

**Figure 1:**
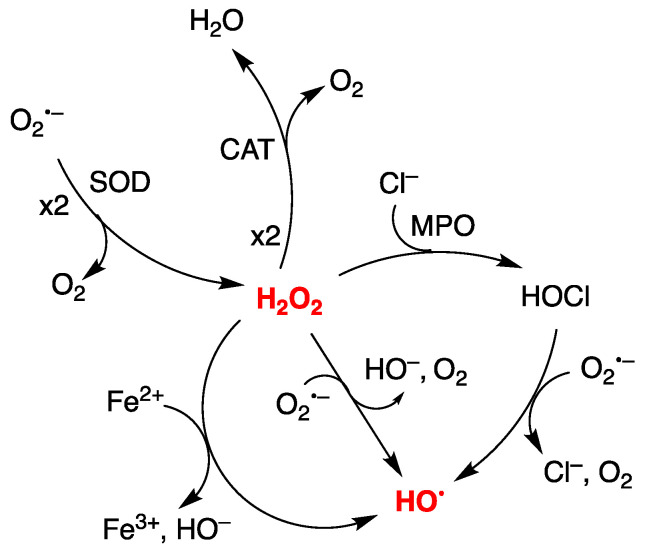
Relevant pathways of the reactive oxygen species (ROS) network: hydroxyl radical (HO^•^) formation from superoxide radical anion (O_2_^•−^) in the mitochondria. SOD: superoxide dismutase; CAT: catalase; MPO: myeloperoxidase.

ROS include radicals such as the superoxide radical anion (O_2_^•−^), nitric oxide (NO^•^), hydroxyl radical (HO^•^), nitrogen dioxide (NO_2_^•^), and the carbonate radical anion (CO_3_^•−^), as well as molecules such as hydrogen peroxide (H_2_O_2_), hypochlorous acid (HOCl), and peroxynitrite (ONOO^−^) [6,21,22]. In quantitative terms, O_2_^•−^ is the most abundant radical formed in aerobic organisms and the main entry to the ROS network. Aerobic life would not be possible without the enzymes known as superoxide dismutases (SODs) and catalase (CAT), which transform O_2_^•−^ to water [23]. As shown in Figure 1, H_2_O_2_ is at the crossroad of several pathways: H_2_O_2_ transformation to highly reactive HO^•^ occurs by the Fenton reaction (Fe^2+^ + H_2_O_2_) and Haber–Weiss reaction (O_2_^•−^ + H_2_O_2_) [22,24]; myeloperoxidase (MPO) uses H_2_O_2_ and Cl^−^ to generate HOCl, which further reacts with O_2_^•−^ to produce HO^•^ [25]. Therefore, O_2_^•−^ being quite unreactive in typical free radical reactions, such as hydrogen atom abstraction or addition, is converted to H_2_O_2_, which is able to diffuse and generate the most reactive HO^•^ radical. The diffusion distance of HO^•^ is very small because of their high reactivity with all types of biomolecules (DNA is not an exception) and, consequently, there is a low probability to be intercepted by antioxidants [26]. HO^•^ is able to react with DNA, causing single strand breaks, abasic sites, DNA–DNA intrastrand adducts, DNA–protein crosslinks, and base damage [27]. Evidence has been provided that in human fibroblasts, mtDNA may be more vulnerable to H_2_O_2_ compared with nDNA, showing a higher frequency of H_2_O_2_–driven lesions in cell culture models, despite it being a well–known H_2_O_2_ scavenging system (cf. Figure 1) [28]. It has been reported that H_2_O_2_ treatment results in strand breaks or abasic sites that are converted to strand breaks [29]. It has been suggested that the relationship between free radicals and mtDNA mutations is not as straightforward as it is often portrayed [17]. Indeed, it has been reported that the absence of oxidative stress induced mutations in the mitochondrial genome may be due to the rapid degradation of oxidized DNA molecules [30]. Consistently, the mechanism of damaged mitochondrial DNA degradation has been recently characterized [31,32].

**Figure 2:**
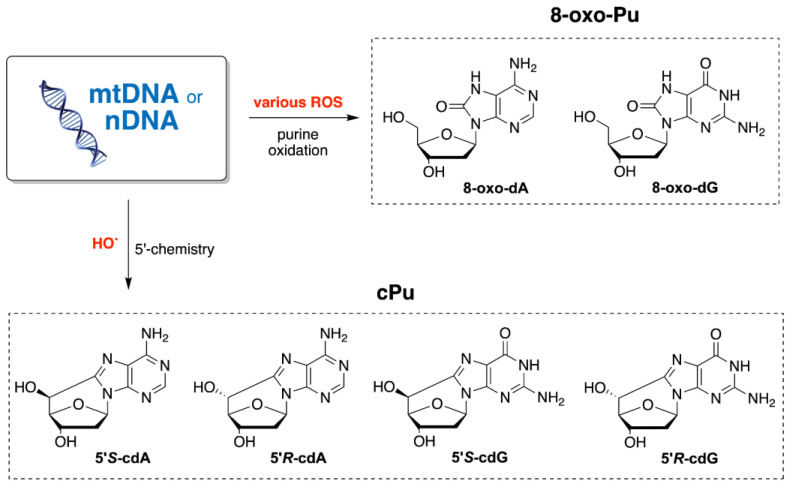
Structures of cPu lesions generated by H–atom abstraction from H5′ position by HO^•^ radical and 8–oxo–Pu lesions generated by purine oxidation from HO^•^ radical and other ROS species.

In the present work, we considered the simultaneous measurement of the six purine lesions in mtDNA shown in Figure 2. 5′,8–cyclopurines (cPu) represent a very interesting and peculiar family of DNA lesions because they are exclusively generated by the reaction of HO^•^ radicals with genetic material via C5′ radical chemistry of the purine moieties [33]. They consist of 5′,8–cyclo–2′–deoxyadenosine (cdA) and 5′,8–cyclo–2′–deoxyguanosine (cdG), existing as 5′*R* and 5′*S* diastereoisomeric forms (Figure 2) [34,35,36]. On the other hand, the 8–oxo–Pu family, which consists of 8–oxo–dG and 8–oxo–dA, is generated by oxidation at the C8 position by a variety of ROS, such as HO^•^ and ROO^•^ radicals, H_2_O_2_, singlet oxygen or ONOO^–^ [33,37]. cPu lesions are substrates of NER, whereas 8–oxo–Pu lesions are substrates of BER [38,39,40,41].

cPu, as transcriptional blocking lesions, have been identified as molecular defects in neurodegenerative processes [42]. In particular, Cockayne syndrome (CS) is an autosomal recessive neurodegenerative premature aging disorder associated with defects in NER. Over 90% of CS cases are due to mutations in either the CSA or CSB genes, responsible for the defect in the transcription coupled nucleotide excision repair (TC–NER) observed in CS cells. The lack of this repair mechanism makes CS cells hypersensitive to UV light. Moreover, cells from CS patients present elevated levels of ROS and are also defective in the repair of a variety of oxidatively generated DNA lesions [43,44,45]. Additionally, elevated levels of mitochondrial DNA damage, hypersensitivity to bioenergetic inhibitors, redox unbalance due to an increase of mitochondrial ROS, and mitochondrial dysfunction have been reported in CS cells [20]. We recently reported two studies on the oxygen–dependent accumulation of purine lesions in total (t) DNA (t = n + mt) [46] and membrane lipidome remodeling [47] in wild type and defective CSA and CSB cell lines. Based on our interest in clarifying the DNA damage scenario, in the present work, we evaluated in this cell system mtDNA damage of purine with a very sensitive protocol (LC–ESI–MS/MS system with isotopomeric internal standards) [33,36,48]. We addressed the following chemical/biological points: (i) the simultaneous measurement of the six purine lesions shown in Figure 2, carried out in the mtDNA of wild type and defective CSA and CSB cell lines, grown under atmospheric oxygen tension; (ii) comparison of the six purine lesions between mtDNA and tDNA, and role of HO^•^ radicals in the oxidatively–induced damage; and (iii) the model reactivity of mtDNA and tDNA in an “isolated” context, using the reaction of genetic material with HO^•^ radicals under biomimetic conditions [49] and measuring the levels of six lesions. The results contribute to a better understanding of the genome integrity features under HO^•^ radical reactivity estimating the contribution of different helical topology in distinct genetic pools such as mtDNA and nDNA.

## 2. Materials and Methods

### 2.1. Cell Lines and DNA Isolation

CSA and CSB SV40–transformed cell lines were established and cultured as previously described [50]. More precisely, an isogenic cell line that expresses the wtCSA protein tagged with the Flag and HA epitopes (CS3BE–wtCSA) was used. The defective counterpart is CS3BE [51]. For CSB cell lines, we used CS1AN–wtCSB and CS1AN (defective CSB cells) [52]. Defective cell lines carry the empty vector. Cell culture studies are grown under standard atmospheric oxygen tension. The mitochondria were isolated by a non–mechanical, reagent–based method according to the procedure of The Mitochondria Isolation Kit (Thermo Fisher Scientific, Waltham, MA, USA). Then, mtDNA was extracted using a high–salt extraction procedure [46,50]. A similar procedure was used for the isolation of tDNA.

### 2.2. γ–Radiolysis Experiments

Each sample of mtDNA and tDNA from CSA1N–wtCSB was dissolved in double distilled water (ddH_2_O) with a concentration of 0.5 mg/mL; in particular, 33.5 μg of tDNA was suspended in 67 μL and 45.9 μg of mt–DNA was suspended in 91.8 μL. The solution was placed in a glass vial of 2 mL containing a 300 µL glass insert, flushed with N_2_O for 10 min and exposed to gamma rays at room temperature (22 ± 2 °C) using a ^60^Co–Gammacell apparatus at different doses (dose rates: 1.44 Gy/min). The exact absorbed radiation dose was determined with the Fricke chemical dosimeter, by taking G(Fe^3+^) 1.61 μmol J^−1^ [53]. The irradiation doses used were 0, 20, and 40 Gy for tDNA and 0, 15, 30, and 45 Gy for mtDNA. The experiments were performed in triplicate. The samples were lyophilized after the irradiation experiments.

### 2.3. Enzymatic Digestion and Quantification of Modified Nucleosides by Stable Isotope LC–MS/MS

**Figure 3:**
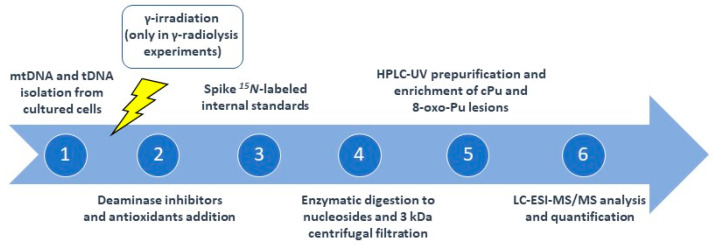
Flow diagram showing protocol steps for the quantification of cPu and 8–oxo–Pu lesions via isotope–dilution LC–ESI–MS/MS.

The purine DNA lesions levels were quantified as described previously [33,36,46,54] and are summarized in Figure 3. Briefly, 10 μg of DNA were enzymatically digested in a reaction mixture including 0.2 mM pentostatin, 5 μM BHT, 3 mM deferoxamine, and the internal standards ([^15^N_5_]–5′*S*–cdA, [^15^N_5_]–5′*R*–cdA, [^15^N_5_]–5′*S*–cdG, [^15^N_5_]–5′*R*–cdG, [^15^N_5_]–8–oxo–dG and [^15^N_5_]–8–oxo–dA), the samples were filtered off by centrifugation through a 3 kDa microspin filter, and were cleaned up and enriched by an HPLC–UV system coupled with a sample collector and injected into the LC–MS/MS system. The quantification of the modified nucleosides was carried out by a triple–stage quadrupole mass spectrometer (Thermo, Waltham, MA, USA) using positive electrospray ionization (ESI) following a gradient program (2 mM ammonium formate, acetonitrile, and methanol) and the detection was executed in multiple reaction monitoring mode (MRM) using the two most intense and characteristic precursor/product ion transitions for each lesion [55,56].

### 2.4. Statistical Analysis

All of the measurements were performed in triplicate and the data were expressed as mean ± standard deviation (SD). The unpaired *t*-test was used for the statistical analysis and a two–tailed *p*-value < 0.05 and *p*-value < 0.01 were considered to indicate a statistically significant difference.

## 3. Results and Discussion

### 3.1. Purine mtDNA Lesions Levels in Wild Type and Defective CSA and CSB Cells

The mtDNA from wtCSA (CS3BE–wtCSA), wtCSB (CS1AN–wtCSB), and defective counterparts CS3BE and CS1AN cell lines cultivated under standard atmospheric oxygen tension have been isolated. After hydrolysis of the genetic material to single nucleosides by an enzymatic cocktail containing nucleases, analysis by liquid chromatography with tandem mass spectrometry (LC–MS/MS) was performed for the determination of the modified nucleosides (four cPu and two 8–oxo–Pu), in accordance with a recently optimized protocol [33,36,46,55,56]. The levels of 8–oxo–dG and 8–oxo–dA are reported in Table 1. Unexpectedly, none of the four cPu lesions were detected. 8–oxo–dG was found to be significantly raised in defective CSB cells compared with the wild type cell line (*p* = 0.011).

Comparing the 8–oxo–dG and 8–oxo–dA levels of mtDNA (Table 1) with the corresponding values of tDNA of the same cellular lines reported recently by us (see Table S2) [46], we observed an increase in lesions in all four cellular lines going from tDNA to mtDNA, e.g., the increase in 8–oxo–dG was ~40% in wt cells and ~10% in defective cells. Similar trends of 8–oxo–dG was previously reported in CS cells by the less sensitivity HPLC–ED detection method [52]. On the other hand, the calculated ratio 8–oxo–dG/8–oxo–dA of 3.9 and 4.8 for CSA and CSB cells, respectively (from the data of Table 1), was very similar to the analogous ratio calculated in the tDNA of the same cellular lines (cf. Table S2), indicating comparable reactivities of the two purine bases (dG and dA) towards the ROS independently of the two genetic pools.

**Figure 4:**
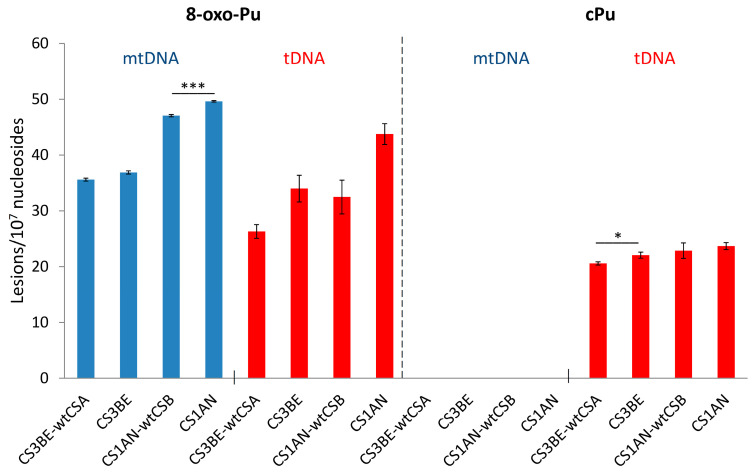
Comparison between 8–oxo–Pu and cPu lesions in the mtDNA and tDNA samples. The levels (lesions/10^7^ Nu) of 8–oxo–Pu and cPu lesions in mtDNA (blue) and tDNA (red) extracted from CS1AN–wtCSB, CS1AN, CS3BE–wtCSA, and CS3BE cells. The error bars represent the standard deviation of the mean, calculated from three independent samples, * denotes a statistically significant difference (*p* < 0.05) between the groups, *** denotes a statistically significant difference (*p* < 0.001) between the groups (see Table S3).

Table 2 shows the levels of total 8–oxo–Pu and cPu in mtDNA in comparison with analogous data calculated for tDNA of the same cellular lines, and Figure 4 also illustrates the overall behavior. The 8–oxo–Pu levels were ~ 40% higher in the mtDNA than the tDNA of the wt cells, whereas the increase was limited to ~10% in defective cells. Table 2 and Figure 4 indicate the absence of cPu lesions in the mtDNA, whereas the cPu levels in tDNA were in the same order of 8–oxo–Pu. The four cPu in the mtDNA were undetectable in defective cells and in the wt counterparts (CSA and CSB). Based on the limit of detection of our analytical methodology, we can infer that the total number of cPu lesions was at least two orders of magnitude lower in the mtDNA compared with the tDNA.

The absence of cPu lesions in the mtDNA in both defective cells and in the wt counterparts (CSA and CSB) may indicate the absence of reactivity with HO^•^ radicals towards mtDNA. The presence of 8–oxo–Pu in the absence of cPu should be informative of the occurrence of the molecular rather than the radical reactivity. Indeed, similar to HO^•^ radicals, other oxidizing species such as H_2_O_2_ or ONOO^−^ are also able to generate 8–oxo–Pu as DNA lesions [29,37]. It is interesting to note that these oxidizing species are increased in CSA defective cells. Treatment with catalase, a H_2_O_2_ scavenger, has shown that high levels of H_2_O_2_ are present in CSA defective cells [57]. Moreover, CSA defective cells are characterized by increased levels of reactive nitrogen species and peroxynitrite, and decreased levels of NO [57,58].

In humans, mitochondrial DNA represents about 1–10% of total cellular DNA (about 1000 to 10,000 copies per cell). In contrast with the invariable copy number of the nuclear genome (diploid), a single cell can contain many copies of mtDNA dependent on different processes involved in the mitochondrial homeostasis, such as mitochondrial replication, mitochondrial dynamics, and mitophagy. It has been estimated that each human cell contains from hundreds to thousand mitochondria [59]. Cells with a higher energy expenditure have a higher number of mitochondria and, consequently, more copies of mitochondrial DNA. A recent study estimated that cardiac and skeletal muscle contained between 4000 and 6000 copies of mtDNA per cell, while the liver, kidney, and lung tissues averaged between 500 and 2000 copies [60].

Previous work in human HeLa cell extracts indicated that cdA and cdG lesions are excised with a similar efficiency by NER and that the *R*–diastereoisomers of both cdA and cdG cause greater distortion of the DNA backbone and are better substrates of NER than the corresponding *S* ones [41,61]. However, the absence of NER in the mitochondria is well documented; therefore, the nonappearance of cPu lesions in the mtDNA cannot be due to their repair [17]. In order to understand the HO^•^ reactivity towards DNA helical topology better, we selectively generated HO^•^ radicals by ionizing irradiations in the presence of isolated mtDNA or tDNA samples from wtCSB cells and carried out the quantification of the six purine lesions as described in the next section.

### 3.2. Hydroxyl Radical–Induced Formation of Purine Lesions: tDNA vs. mtDNA

The findings in the cell cultures motivated our interest to investigate the reactivity of mtDNA and tDNA taken out of their biological contexts. HO^•^ radicals are known for their reactivity and ability to cause chemical modifications to DNA, the site of attack being both the base moieties (85–90%) and the 2–deoxyribose units [62]. Therefore, we exposed tDNA and mtDNA, isolated from CS1AN–wtCSB, to HO^•^ radicals generated by irradiation.

Radiolysis of neutral water leads to the reactive species e_aq_^−^, HO^•^, and H^•^ as shown in Reaction 1, together with H^+^ and H_2_O_2_. The values in parentheses represent the radiation chemical yields (*G*) in units of μmol J^−1^. In an N_2_O–saturated solution (~0.02 M of N_2_O), e_aq_^−^ are converted into the HO^•^ radical via Reaction 2 (*k*_2_ = 9.1 × 10^9^ M^−1^ s^−1^), with *G*(HO^•^) = 0.55 μmol J^−1^, i.e., HO^•^ radicals and H^•^ atoms account for 90 and 10%, respectively, of the reactive species [63,64]. The rate constants for the reactions of HO^•^ radicals and H^•^ atoms with DNA (Reactions 3 and 4) have been reported to be ca. 2.5 × 10^8^ M^−1^ s^−1^ and 6 × 10^7^ M^−1^ s^−1^, respectively [62,63].

H_2_O + *ɣ–irr* → e_aq_^−^(0.27), HO^•^(0.28), H^•^(0.062), H^+^(0.27), H_2_O_2_(0.07) (1)

e_aq_^−^ + N_2_O + H_2_O → HO^•^ + N_2_ + HO^−^ (2)

HO^•^ + DNA → radical product (3)

H^•^ + DNA → radical product (4)

**Figure 5:**
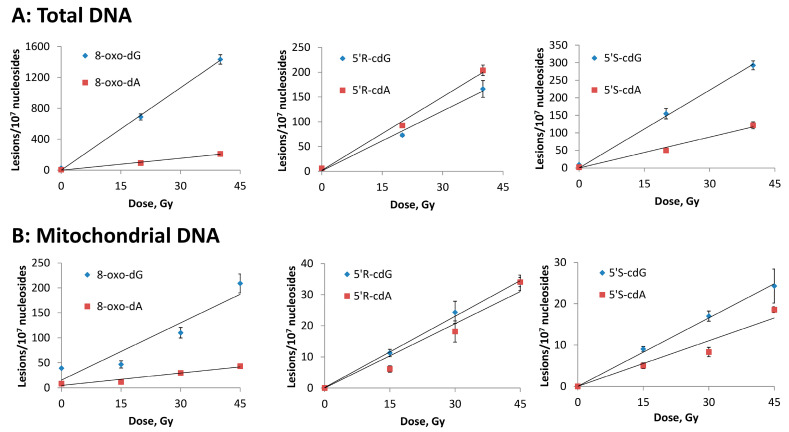
γ–Irradiation induced formation of 8–oxo–dG, 8–oxo–dA, 5′*R*–cdG, 5′*R*–cdA, 5′*S*–cdG, and 5′*S*–cdA in (**A**) tDNA and (**B**) mtDNA samples; the values represent the mean ± SD of *n* = 3 independent experiments.

Figure 5 summarizes our findings and the resulting formation of 8–oxo–dG, 8–oxo–dA, 5′*R*–cdG, 5′*R*–cdA, 5′*S*–cdG, and 5′*S*–cdA in the function of irradiation doses in both tDNA and mtDNA (for specific values, see Table S4). As expected, the number of the lesions studied increased with the increment of the dose [55,56]. 8–oxo–dG is the main detected lesion, whereas 8–oxo–dA is formed in lower yields and similarly for each of the four cPu (5′*S*–cdG, 5′*R*–cdG, 5′*S*–cdA and 5′*R*–cdA). Furthermore, the slope of the obtained lines represents the number of lesions formed per Gy, as summarized in Table 3 (cf. Table S5).

From the analysis of the data reported in Table 3 in terms of lesions/10^7^ nu/Gy, the ratios 8–oxo–dG/8–oxo–dA were 6.9 and 4.8 in tDNA and mtDNA, respectively. It is worth mentioning that the same ratio in calf–thymus DNA was found to be 7.7 under similar experimental conditions, although the number of lesions/Gy was four to five times higher with respect to tDNA [55]. The mechanism of the formation of 8–oxo–dG through the reaction of HO^•^ radical with ds–oligonucleotide [55,56] and calf–thymus DNA [65,66] has been investigated in detail. It was demonstrated that the addition of HO^•^ to the C8 position of the guanine moiety accounts for a minor percentage (~10%), whereas the main yield of 8–oxo–dG is produced by a one–electron oxidation reaction involving DNA radicals [56,65,66]. The lower yield of formation of 8–oxo–dA was attributed, to a minor extent, to the latter path in the case of adenine moieties. However, the HO^•^–adducts of guanine and adenine moieties afforded a variety of products, including 8–oxo–dG and 8–oxo–dA [33,67]. It is worth underlining that the lesions/Gy decreased substantially when going from tDNA to mtDNA (9.3 times for 8–oxo–dG and 6.4 times for 8–oxo–dA, see Table 3).

**Scheme 1:**
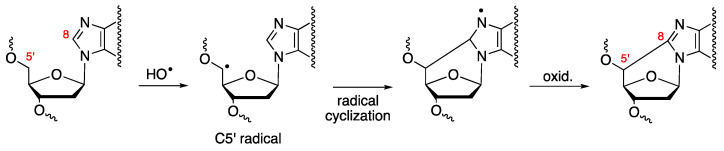
Purine 2′–deoxynucleoside reacts with HO^•^ yielding cPu via the cyclization of the C5′ radical followed by oxidation.

Intramolecular cyclization products 5′*R*–cdG, 5′*R*–cdA, 5′*S*–cdG, and 5′*S*–cdA were also formed in the same fashion, but in lower yields (Figure 4). The slope of the lines obtained represents the number of lesions formed per Gy, and these data are also reported in Table 3. The attack at the H5′ of DNA by HO^•^ was estimated to be 55% for all possible sugar positions and the resulting C5′ radical in the purine nucleotide moieties likely evolved with an internal cyclization onto the C8 position of the base with the formation of cPu as the final product (Scheme 1) [33,34,35,36]. The most important finding was the significant reduction in lesions/Gy going from tDNA to mtDNA (5.0, 6.1, 14.2, and 7.5 times for 5′*R*–cdG, 5′*R*–cdA, 5′*S*–cdG, and 5′*S*–cdA, respectively), similar to the case of 8–oxo–dG and 8–oxo–dA (9.3 and 6.4 times, respectively). The observed differences between tDNA and mtDNA in the reaction with HO^•^ were likely due to the different topology of the nuclear and mitochondrial genome [68].

**Figure 6:**
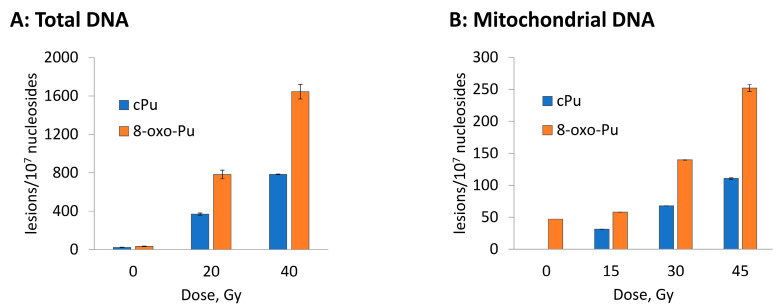
Total amount of cPu and 8–oxo–Pu lesions (lesions/10^7^ nu) in irradiated samples of tDNA (**A**) and mtDNA (**B**). The numbers represent the mean value (± standard deviation) in triplicate.

We also considered the total amount of 8–oxo–Pu and cPu lesions (lesions/10^7^ nu) in both the tDNA and mtDNA of CS1AN–wtCSB cells irradiated at different doses. The scale referring to tDNA (Figure 6A) was six times higher than the scale referring to mtDNA (Figure 6B), clearly indicating the different amounts of accumulated lesions in the two genetic materials (Table S6). The 8–oxo–Pu was nearly twice that of the cPu values in both genomic materials (Figure 6): in units of lesions/10^7^ nu/Gy, we calculated 40.3 for 8–oxo–Pu and 19.0 for cPu in tDNA, and 4.6 for 8–oxo–Pu and 2.5 for cPu in mtDNA. These values also indicate that 8–oxo–Pu and cPu were 8.8 and 7.6 times, respectively, higher in the tDNA than in the mtDNA.

From the reaction of the HO^•^ radicals with tDNA and mtDNA, it is clear that (i) the formation 8–oxo–Pu was twice that of cPu, indicating a true competition of the two pathways in Figure 2, although we cannot exclude a small percentage of 8–oxo–Pu formation by the reaction of H_2_O_2_ generated from the irradiation of water (see reaction (1)), and (ii) the values of both 8–oxo–Pu and cPu in tDNA were ~8 times higher than in mtDNA, revealing a different accessibility of HO^•^ to mtDNA and nDNA structures. The structure of mtDNA is arranged in a loop, which is loosely supercoiled [12]. The comparison of 8–oxo–Pu lesions arising from γ–radiation sourced HO^•^ on tertiary DNA helical forms of supercoiled (SC), open circular (OC), and linear (L) conformation was known [68]. Purine oxidation in dsDNA follows L > OC >>SC, indicating increased damage towards the extended B–DNA topology, where 8–oxo–dG and 8–oxo–dA levels increase ≥10–fold in both circular and linear conformers.

**Figure 7:**
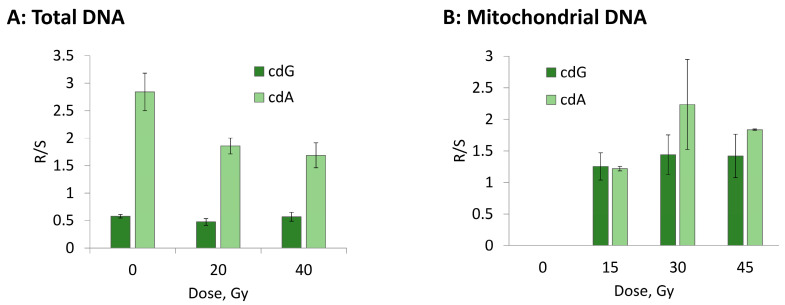
Diastereoisomeric ratios (5′*R*/5′*S*) for cdG and cdA lesions in irradiated samples of tDNA (**A**) and mtDNA (**B**). The numbers represent the mean value (± standard deviation) in triplicate.

The cPu lesions can be present in two diasteroisomeric forms, 5′*R* and 5′*S*, for each 2′–deoxyadenosine and 2′–deoxyguanosine moieties. The diastereomeric ratio (*R*/*S*) attracts interest as it can inform on mechanistic issues that are related to structural conformations of both isomers in association with their abundance. Figure 7 shows the *R*/*S* ratios for cdG and cdA lesions of irradiated tDNA and mtDNA samples. The reported data indicate that the ratios are similar within tDNA or mtDNA and independent of the irradiation dose, i.e., the *R*/*S* average ratios are 0.52 for cdG and 1.75 for cdA in tDNA, whereas in mtDNA they are 1.35 for cdG and 1.74 for cdA (Table S7).

It is worth underlining that in previous studies using extracted tDNA from various animal tissues, the *S* form was found to always be more abundant than the *R* form in cdG, whereas in cdA, the *R* form was always more abundant than the *S* form [36], this is in accordance with our present results. For example, the *R/S* levels of the cdG and cdA lesions in the tDNA of the liver and kidney of normal Swiss mice associated with age–related processes [69] are similar to the values of Figure 7A. In the brain of a rat model of Wilson disease, the *S*-cdG was always higher than the *S*-cdA (1.5 times) [70] similar to the ratios in the brains of normal, SCID, and tumor–bearing mice [71]; in the latter, the *R/S* cdA was always higher that the cdG ratio (approximately 1.3-1.6 times). Moreover, in various tissues (such as the brain, spleen, and liver) of prdx1^–/–^ mice, the *R/S* cdG was found to be 0.23, 0.14, and 0.40 while the *R/S* cdA was 0.11, 0.23, and 0.08 [72]. As Figure 6B shows, in the mtDNA in both cdG and cdA, the *R* form is always more abundant in both cdG and cdA. In this respect, it is worth recalling that the *R/S* ratios of 8.3 for cdG and 6 for cdA were obtained in water upon the irradiation of free nucleosides (*R* form is always more abundant) [73,74], indicating that the diastereomer ratio is dependent on the molecular complexity. The *R/S* ratios can be used to support further biological implications in the formation and/or repair of these lesions, although a clear scenario for the *R/S* formation is still missing [36]. The structure of mtDNA is arranged in a loop, with one strand called H (heavy; purine rich) and the other strand called L (light; pyrimidine rich) [12]. We are suggesting that this arrangement strongly influences the local conformations at the reactive sites prior to C5′ radical cyclization, which makes one type of diastereoisomer more prevalent.

## 4. Conclusions

In this work, we measured the purine lesions in mtDNA of four cellular lines, i.e., wtCSA, wtCSB, and their defective counterparts, and compared with their analogous data of tDNA. The 8–oxo–Pu lesions were comparable in mtDNA and tDNA, although they were found to be constantly higher in mtDNA for all four cellular lines. The cPu lesions were undetectable in mtDNA, suggesting at least a 100 times lower level than in tDNA. We evidenced, for the first time, the DNA damage scenario as a contribution of two distinctive pathways, i.e., the molecular ROS and the radical ROS species, which can be clearly distinguished by comparing our results in the cell cultures with the cPu levels of the irradiation experiments. Indeed, we evaluated that the absence of cPu lesions in the mtDNA of the cellular experiment may indicate a nonappearance of HO^•^ radical reactivity and that the amount of 8–oxo–Pu in mtDNA was consistent with the contribution of DNA reactivity with oxidizing species, such as H_2_O_2_ or ONOO^−^. Moreover, a suggestive hypothesis to explain the absent accumulation of cPu adducts in the mtDNA of CS cells could play a crucial role in specific mechanisms devoted to the maintenance of the mitochondrial genome integrity. In particular, in mammalian cells, a degradation mechanism of damaged mtDNA molecules has been recently discovered [32]. Moreover, mitophagy and mitochondrial fission can further contribute to the removal of dysfunctional/damaged organelles reducing the cPu levels up to undetectability. In the reaction of γ–irradiation generated HO^•^ radicals with isolated tDNA and mtDNA, the values of all six purine lesions in the tDNA are ~8 times higher than in mtDNA. We evidenced a different accessibility of HO^•^ into mtDNA and nDNA structures which were associated with different helical topologies.

## Figures and Tables

**Table 1:** The levels (lesions/10^7^ nucleosides) of 8–oxo–dG and 8–oxo–dA in mtDNA isolated from CSA and CSB (wt and defective) cells.

mtDNA	8–oxo–dG ^1^	8–oxo–dA ^1^
CS3BE–wtCSA	28.30 ± 0.13	7.32 ± 0.13
CS3BE	29.31 ± 0.22	7.58 ± 0.07
CS1AN–wtCSB	39.00 ± 0.18 *	8.08 ± 0.15
CS1AN	40.90 ± 0.22 *	8.71 ± 0.17

^1^ The numbers represent the mean value (±standard deviation) of the DNA lesions levels from the measurement of triplicate; statistical significance: * (*p* < 0.05) was observed between CS1AN–wtCSB and CS1AN cell samples (see Table S1).

**Table 2:** Total amount of 8–oxo–Pu and cPu (lesions/10^7^ nucleosides) in mtDNA in comparison with values of tDNA isolated from CSA and CSB (wt and defective) cells.

	8–oxo–Pu ^1^		cPu ^1^	
	mtDNA ^2^	tDNA ^3^	mtDNA ^2^	tDNA ^3^
CS3BE–wtCSA	35.62 ± 0.26	26.32 ± 1.22	N/D	20.56 ± 0.30 *
CS3BE	36.89 ± 0.28	33.98 ± 2.40	N/D	22.04 ± 0.55 *
CS1AN–wtCSB	47.08 ± 0.21 ***	32.49 ± 3.04	N/D	22.84 ± 1.39
CS1AN	49.61 ± 0.13 ***	43.73 ± 1.86	N/D	23.67 ± 0.62

^1^ The numbers represent the mean value (± standard deviation) of DNA lesions levels from the measurement of triplicate. ^2^ Present work; N/D = not detected. ^3^ From [46]. Statistically significant samples * (CS1AN–wtCSB vs. CS1AN; *p* = 0.035); *** (CS1AN–wtCSB vs. CS1AN; *p* = 0.0005).

**Table 3:** The levels of 8–oxo–dG, 8–oxo–dA, 5′*R*–cdG, 5′*R*–cdA, 5′*S*–cdG, and 5′*S*–cdA (Lesions/10^7^ nu/Gy) from the irradiation of N_2_O saturated tDNA or mtDNA (0.5 mg/mL) of CS1AN–wtCSB in aqueous solutions.

Lesion	tDNA	mtDNA
8–oxo–dG	35.2	3.8
8–oxo–dA	5.1	0.8
5′*R*–cdG	4.0	0.8
5′*R*–cdA	4.9	0.8
5′*S*–cdG	7.1	0.5
5′*S*–cdA	3.0	0.4

## Data Availability

The data presented in this study are available in this article and the Supplementary Material.

## Supplementary Materials

The following supporting information can be downloaded at: https://www.mdpi.com/article/10.3390/biom12111630/s1, Table S1: The levels of 8–oxo–dG, 8–oxo–dA, 5′*R*–cdG, 5′*S*–cdG, 5′*R*–cdA, and 5′*S*–cdA lesions in mtDNA isolated from CSA and CSB (wt and defective) cells; Table S2 and Table 3: Statistical analysis by comparing the means of lesions in mtDNA isolated from CSA and CSB cells (wt vs. defective); Table S4: Total amount of 8–oxo–dG, 8–oxo–dA, 5′*R*–cdG, 5′*R*–cdA, 5′*S*–cdG, and 5′*S*–cdA (lesions/10^7^ nu) from the of N_2_O saturated tDNA or mtDNA of CS1AN–wtCSB in aqueous solutions; Table S5: Equation and R–squared value of Figure 5 plots; Table S6: The levels of cPu and 8–oxo–Pu lesions from the irradiation experiments; Table S7: 5′*R*/5′*S* ratio for both cdG and cdA from the irradiation experiments.
