# Supplementary material for: Assessing the Formation of Purine Lesions in Mitochondrial DNA of Cockayne Syndrome Cells
Source: Biomolecules. 2022 Nov 3;12(11):1630. doi: 10.3390/biom12111630 (PMC9687895; doi:10.3390/biom12111630)
Supplement: Supplementary file 1 [file biomolecules-12-01630-s001.zip › biomolecules-1966527-supplementary.pdf]

Supplementary Material

Assessing the Formation of Purine Lesions in Mitochondrial DNA of Cockayne Syndrome Cells

Chryssostomos Chatgilialoglu *, Marios G. Krokidis, Annalisa Masi, Sebastian Barata-Vallejo, Carla Ferreri, Barbara Pascucci and Mariarosaria D'Errico

* Correspondence: chrys@isof.cnr.it

Table S1: Values of *t*-Test (Two-Sample Assuming Unequal Variances) by comparing lesions in mtDNA isolated from CSA and CSB cells (wt vs. defective).

	**8-oxo-dG**	**8-oxo-dA**
CS3BE-wtCSA vs CS3BE	0.149	0.319
CS1AN-wtCSB vs CS1AN	0.011*	0.062

Statistically significant samples: * ($p < 0.05$)

Table S2. The levels (lesions/10^7 nucleosides) of 8-oxo-dG and 8-oxo-dA in tDNA isolated from CSA and CSB (wt and defective) cells (Taken from reference [46]).

**tDNA**	**8-oxo-dG** [1]	**8-oxo-dA** [1]
CS3BE-wtCSA	20.82 ± 1.44	5.49 ± 0.56*
CS3BE	26.49 ± 3.03	7.49 ± 0.74*
CS1AN-wtCSB	26.78 ± 3.30	5.72 ± 0.28
CS1AN	37.55 ± 1.19	6.18 ± 0.94

[1] The numbers represent the mean value (± standard deviation) of DNA lesions levels from the measurement of triplicate; statistical significance: * ($p = 0.040$) was observed between CS3BE-wtCSA and CS3BE cell samples.

Table S3. Values of *t*-Test (Two-Sample Assuming Unequal Variances) by comparing lesions in mtDNA isolated from CSA and CSB cells (wt vs. defective).

	**8-oxo-Pu**
CS3BE-wtCSA vs CS3BE	0.187
CS1AN-wtCSB vs CS1AN	0.0005***

Statistically significant samples: *** ($p < 0.001$)

Table S4. Total amount of 8-oxo-dG, 8-oxo-dA, 5'*R*-cdG, 5'*R*-cdA, 5'*S*-cdG and 5'*S*-cdA (lesions/10^7 nu) from the irradiation of N_2O saturated tDNA or mtDNA (0.5 mg/mL) in aqueous solutions. [1]

**Sample**	**Dose, Gy**	**8-oxo-dG**	**8-oxo-dA**	**5'*R*-cdG**	**5'*R*-cdA**	**5'*S*-cdG**	**5'*S*-cdA**
tDNA	0	26.78 ± 3.30	5.72 ± 0.28	5.25 ± 0.53	6.28 ± 0.15	9.08 ± 0.68	2.23 ± 0.26
	20	688.68 ± 39.23	93.17 ± 5.39	72.98 ± 2.56	92.58 ± 2.81	154.44 ± 14.95	49.92 ± 2.37
	40	1433.93 ± 61.62	210.69 ± 14.55	166.14 ± 16.80	203.75 ± 10.57	292.53 ± 12.58	121.48 ± 10.00
mtDNA	0	39.00 ± 0.18	8.08 ± 0.15	N/D [2]	N/D [2]	N/D [2]	N/D [2]
	15	46.51 ± 7.09	11.45 ± 1.83	11.26 ± 1.19	6.10 ± 1.02	9.04 ± 0.61	5.00 ± 0.69
	30	110.05 ± 10.40	29.55 ± 2.91	24.31 ± 3.58	18.17 ± 3.40	17.00 ± 1.22	8.31 ± 1.13
	45	208.91 ± 19.00	43.18 ± 2.14	33.79 ± 2.51	34.05 ± 1.51	24.28 ± 4.12	18.55 ± 0.73

[1] The values are the mean value (± standard deviation) of DNA lesions levels from the measurement in triplicate. [2] N/D = non detected.

Table S5. Equation and R-squared value of Figure 5 plots

**tDNA**				**mtDNA**		
**lesion**	**equation**	**R^2**		**lesion**	**equation**	**R^2**
8-oxo-dG	y = 35.17x + 12.88	0.998		8-oxo-dG	y =3 .821x + 15.12	0.886
8-oxo-dA	y = 5.124x + 0.704	0.992		8-oxo-dA	y = 0.822x + 4.551	0.944
5'R-cdG	y = 4.022x + 1.01	0.991		5'R-cdG	y = 0.762x + 0.177	0.996

5'R-cdA	y = 4.936x + 2.137	0.994		5'R-cdA	y = 0.761x - 2.553	0.964
5'S-cdG	y= 7.086x + 10.29	0.999		5'S-cdG	y = 0.538x + 0.458	0.997
5'S-cdA	y = 2.981x - 1.749	0.986		5'S-cdA	y = 0.393x - 0.879	0.942

Table S6. Total amount of cPu and 8-oxo-Pu (lesions/10^7 nucleosides) in tDNA and mtDNA samples (taken from Table S4).

Sample	Dose, Gy	8-oxo-Pu	cPu
tDNA	0	32.49 ± 3.04	22.83 ± 1.38
	20	781.85 ±44.62	369.93 ±11.95
	40	1644.62 ±76.17	783.90 ±3.65
mtDNA	0	47.08 ±0.20	0
	15	57.95 ±5.26	31.40 ±1.12
	30	139.60 ±13.31	67.79 ±4.63
	45	252.08 ±21.13	110.68 ±0.62

Table S7. Diastereoisomeric ratios (5'*R*/5'*S*) for both cdG and cdA in tDNA and mtDNA samples (taken from Table S4).

Sample	Dose, Gy	*R*/*S* cdG	*R*/*S* cdA
tDNA	0	0.58 ± 0.03	2.84 ± 0.34
	20	0.48 ± 0.06	1.86 ± 0.14
	40	0.57 ± 0.08	1.69 ± 0.23
mtDNA	0	0	0
	15	1.25 ± 0.22	1.22 ± 0.04
	30	1.44 ± 0.31	2.23 ± 0.71
	45	1.42 ± 0.34	1.84 ± 0.01
